# Supplementary material for: Nested order-disorder framework containing a crystalline matrix with self-filled amorphous-like innards
Source: Nat Commun. 2022 Aug 9;13:4650. doi: 10.1038/s41467-022-32419-5 (PMC9363411; doi:10.1038/s41467-022-32419-5)
Supplement: Supplementary file 1 — Supplementary Information [file 41467_2022_32419_MOESM1_ESM.pdf]

Supplementary Information

Nested order-disorder framework containing a crystalline matrix with self-filled amorphous-like innards

Kejun Bu,[1] Qingyang Hu,[1] Xiaohuan Qi,[2] Dong Wang,[1] Songhao Guo,[1] Hui Luo,[1] Tianquan Lin,[2] Xiaofeng Guo,[3] Qiaoshi Zeng,[1] Yang Ding,[1] Fuqiang Huang,[2] Wenge Yang,[1] Ho-Kwang Mao[1] & Xujie Lü[1,*]

[1] *Center for High Pressure Science and Technology Advanced Research, Shanghai 201203, China*

[2] *CAS Key Laboratory of Materials for Energy Conversion, Shanghai Institute of Ceramics, Chinese Academy of Sciences, Shanghai 200050, China*

[3] *Department of Chemistry and Alexandra Navrotsky Institute for Experimental Thermodynamics, Washington State University, Pullman, WA 99164, United States*

[*]Corresponding author: Xujie Lü (xujie.lu@hpstar.ac.cn)

List of contents:

1. Supplementary Methods

    1.1 X-ray crystallography

    1.2 Thermal conductivity measurement

    1.3 Density Functional Theory (DFT) calculations

    1.4 First-Principles Molecular Dynamics (FPMD) simulation

2. Supplementary Discussion

    2.1 In situ Raman spectroscopy

    2.2 Optical property

    2.3 Electron transport property

3. Supplementary Figures and Tables
4. Supplementary References

1. Supplementary Methods

1.1 X-ray crystallography

The in situ single-crystal XRD at high pressures was carried out at the experimental station 13 BM-C (GSECARS) of Advanced Photon Source (APS), Argonne National Laboratory (ANL). Silicon oil was used as pressure transmitting medium in the single-crystal XRD experiments.[1] The X-ray beam was monochromated with silicon 311 crystal to 28.6 keV (0.434 Å), with 1 eV bandwidth. A Kirkpatrick-Baez mirror system was used to obtain a vertical × horizontal focus spot size of 15 μm × 15 μm, measured as the full width at half maximum (FWHM). The MAR165 Charge Coupled Device (CCD) detector (Rayonix) was placed about 160 mm away from the sample, and the ambient LaB_6 powder was used to calibrate the distance and tilting of the detector. The sample was placed on the rotation center of the diffractometer and was aligned by an optical microscope. For high pressure diffraction, the angular range of the wide rotation exposure was φ = -15° to φ = 15°, limited by the maximum opening angle of the diamond anvil cell, and followed by a series of step φ-exposures, each covering 0.5° scan width. The typical exposure time was 1 s/°.

In situ powder XRD experiments were performed at beamline 16 ID-B of HPCAT at APS, ANL. Neon was used as the pressure transmitting medium in the powder XRD experiments. The wavelength of the monochromatic X-ray beam is 0.4066 Å (30.5 keV). The diffraction patterns were collected by the MarCCD detector and integrated using the Dioptas software.[2] Structure refinements were carried out by using the Rietveld method in FullProf software.[3] The XRD result collected at ambient pressure confirms the pure phase of our sample and the lattice constant (*I*-43*m*, *a* = 10.368 Å) is consistent with the report from Wuensch, B. J.[4] Then, we used the cubic phase as the starting parameters to refine structures ranging from 0 to 16.9 GPa.

The quantity of compressibility is conventionally defined as the relative change rate of dimension with pressure,

$$K_i = -\left(\frac{1}{i}\right)\left(\frac{\partial i}{\partial p}\right) \qquad \text{(Supplementary Equation 1)}$$

where i can be assigned as V (volume), A (area), and l (length) for volume, area, and linear compressibility, respectively.[5]

1.2 Thermal conductivity measurement

To measure the pressure dependence of thermal conductivity (κ), we developed a technique based on high-pressure Raman scattering measurements by changing temperatures and laser powers. In our measurements, neon was used as the pressure transmitting medium. The heat dissipation through the neon medium can be safely neglected due to its low κ.[6] Meanwhile, the sample was chosen a large single crystal to avoid the boundary heat (Fig. 4c inset). A similar method has been used to determine the κ of thin films, such as MoS_2 and graphene.[7, 8] The focused laser beam will introduce a localized heating effect on the cuboid sample, which has been observed previously.[9] Within the ideal thermal transport model shown in Supplementary Fig. 10, κ can be expressed as:[8]

$$\kappa \frac{1}{r}\frac{d}{dr}\left[\frac{1}{r}\frac{T_{\mathrm{r}}}{r}\right] + q(r) = 0 \qquad \text{(Supplementary Equation 2)}$$

where T_{r} is the temperature distribution, r is the position measured from the heating point, and $q(r)$ is the heat flux distribution. The boundary thermal transport and heat dissipation are neglected in such a model, and the isothermal surface is hemispheric.

The calculated total thermal conductivity is the sum of the lattice (κ_{L}) and electronic (κ_{e}) components.[10] It is very difficult to distinguish the electronic and lattice contributions to thermal conductivity at high pressures. However, a quantitative estimation of such contributions in $Cu_{12}Sb_4S_{13}$ can be given with the knowledge of pressure dependences of electrical resistivity (ρ) and the FWHM of the Raman modes.

The lattice thermal conductivity κ_{L} is approximately proportional to the relaxation time for the phonon transport behavior in crystals.[11] The relaxation time τ for a certain optical mode can be described by the FWHM of the phonon peak as:[11]

$$\tau = \frac{1}{2\pi \mathrm{FWHM}} \qquad \text{(Supplementary Equation 3)}$$

Thus, we use the following equation to calculate κ_{L}:[12]

$$\kappa_L = \frac{A}{2\pi FWHM} \qquad \text{(Supplementary Equation 4)}$$

Having κ_L and the FWHM value for the Raman modes at ambient conditions, we determined the parameter A. Assuming that A is not a pressure-sensitive parameter, we can obtain the evolution of the κ_L with pressure based on the FWHM behavior of the Raman modes. The optical modes of $Cu_{12}Sb_4S_{13}$ are Raman active.[11] The Sb−S stretching (A_1) optical mode is related to the variation of lattice contraction.[13] Thus, the FWHM values of A_1 mode can be used to estimate the κ_L.

The electronic thermal conductivity κ_e is determined by the Wiedemann-Franz law:[10]

$$\kappa_e = LT/\rho \qquad \text{(Supplementary Equation 5)}$$

with L being the Lorenz number, is believed to provide a good description of the relationship between κ_e and electrical resistivity ρ for metals or semiconductors. The Lorenz number L is equal to the Sommerfeld value $L_0 = (\pi^2/3)(k_B/e)^2 = 2.44 \times 10^{-8}$ W·Ω·K^{-2}, k_B is the Boltzmann constant and e is the electron charge.

The calculated total thermal conductivity κ is the sum of the lattice and electronic components:[10]

$$\kappa = \kappa_e + \kappa_L \qquad \text{(Supplementary Equation 6)}$$

1.3 Density Functional Theory (DFT) calculations

Density Functional Theory (DFT) calculations were performed using the Vienna Ab Initio Simulation Package (VASP).[14] The Perdew-Burke-Ernzehof (PBE) version of the generalized gradient approximation (GGA) was used to describe the exchange-correlation functional, and the projector augmented wave (PAW) method was used in the present work.[15] Here, the cutoff energy of the plane wave was chosen at 450 eV. For the structure optimizations, 8 × 8 × 8 k-points were used for the conventional cell (Supplementary Table 2). The convergence criteria are the changes that total energies between two successive electronic steps are less than 10^{-5} eV and all the Hellmann-Feynman forces acting on each atom are less than 0.01 eV /Å. The high-symmetry points in the Brillouin zones were considered in our band structure calculations. The following paths are H (0 1 0) − Γ (0 0 0) − P (0.5 0.5 0.5) − PA (0.5 0.5 -0.5) − N (0.5 0.5 0) − Γ (0 0 0) in the cubic phase.

The effective masses of electron are derived from the following expression:

$$m^* = \hbar^2 \left[\frac{\partial^2 \varepsilon(k)}{\partial k^2}\right]^{-1} \qquad \text{(Supplementary Equation 7)}$$

where k is the wave vector along the transport direction, $\varepsilon(k)$ represents the energy band eigenvalues, and $\hbar$ is the reduced Planck constant.

1.4 First-Principles Molecular Dynamics (FPMD) simulation

Before implementing the FPMD simulation, we first relaxed the lattice of $Cu_{12}Sb_4S_{13}$ to the target pressures of 1 bar, 9.4 GPa, and 13.3 GPa. Using the VASP,[16] the structural optimization was performed by employing the generalized gradient approximation functionals parameterized by PBE.[15] We treated 11 electrons ($4s3d^{10}$) for Cu, 5 electrons ($5s^2$ $5p^3$) for Sb atoms, and 6s ($3s^2 3p^4$) electron for S atoms as valence electrons respectively. In a single unit-cell of 58 atoms (24 Cu, 8 Sb, 26 S), the plane-wave basis sets a cutoff energy of 450 eV and k-point sampling of 0.06 $Å^{-1}$. The setups were used to converge the structure such that the interatomic forces were less than 0.01 eV/Å at target pressures. Supercells for FPMD simulations were built based on those optimized structures.

The same pseudopotential and PBE functional were employed in the FPMD simulation. Here, a single gamma-point (Γ) was adopted for k-points sampling molecular dynamics. We doubled the x-axis of the lattice to construct a 2×1×1 supercell, which contains a total number of 116 atoms. The simulation ran under a constant number of atoms, volume, and temperature (NVT) ensemble, as well as a constant number of atoms, pressure, and temperature (NPT) ensemble. Along the trajectory, we now initialize simulation with 3 ps NVT simulation for heating (near 0 K to 300-500 K), with 1 fs for each step and temperature controlled by a Nosé-Hoover thermostat,[17] then follow by 5 ps NPT to the target pressure and eventually run 10 ps NVT simulation for equilibrium. The standard deviations of pressure are generally less than 1 GPa. Reaching equilibrium generally takes 10^4 FPMD steps (10 ps), which is judged by the fluctuation of thermodynamical variables.

2. Supplementary Discussion

2.1 In situ Raman spectroscopy

The phase transition from crystalline to NOF structure is further confirmed by in situ Raman spectroscopy. As shown in Supplementary Fig. 3, two Raman active modes are observed at wavenumbers of 320 and 355 cm^{-1}, corresponding to Sb−S stretching (A_1) and bending (E) modes, respectively.[13] Upon compression, all Raman peaks shift to higher wavenumbers and eventually become unobservable at 14 GPa, which is owing to the bond shortening and lattice disordering of structures, respectively. It is worth noting that the peak intensity and the slope of Raman shift for bending modes (E) obviously increase beyond 6.5 GPa, again indicating the suppression of LPEs changes S−Sb−S bond angles, which is in line with the XRD results.

2.2 Optical property

In situ UV-Vis-NIR absorption spectroscopy was conducted to trace the variation of the bandgap under pressure (Supplementary Fig. 13a). At ambient conditions, the band edge of $Cu_{12}Sb_4S_{13}$ was determined to be 1.23 eV, in line with the previous report.[18] The bandgap increases from 1.23 eV to 1.33 eV during compression, which is attributed to the suppression of the Sb-5s LPEs (Supplementary Fig. 13b). The gradual weakening of the Sb-5s states pulls down the valence band resulting in abnormal blueshift.[19] Eventually, the band edge becomes unobservable beyond 10 GPa.

2.3 Electron transport property

The detailed results of the resistivity ρ of $Cu_{12}Sb_4S_{13}$ at selected pressures are summarized in Fig. 4a. The ρ decreases with increasing pressure and reaches 1.27×10^{-5} Ω·m around 7 GPa, which significantly contributes to κ_e. Then ρ increases sharply with further increasing pressure up to 13 GPa, which is ascribed to the LPEs-induced high vibration of Cu2 sublattices. Such enhanced vibration significantly leads to

lattice scattering and inhibits charge transport. Further compression decreases the resistivity due to the pressure-induced bandwidth broadening and eventual metallization, reaching a low value of ~8×10^{-6} Ω·m at 23.2 GPa. The dramatic change of ρ is controlled by the electronic structure, which can be tuned by pressure. Theoretical calculations reveal the indirect bandgap nature of $Cu_{12}Sb_4S_{13}$ (Supplementary Fig. 15). By applying the parabolic band approximation, we calculated the effective masses of the electron in $Cu_{12}Sb_4S_{13}$ at selected pressures using Supplementary Eq. (7). The effective masses of the electron decrease upon compression (1.61 $m_0 \rightarrow 0.64$ m_0 from 0 to 9.4 GPa), which indicates enhanced carrier mobility (Supplementary Fig. 15). Because of the rising carrier mobility, the electrical conductivity increases up to 7 GPa. However, due to the part-disordered structural transition, electron scattering enhances and brings the decrease of electrical conductivity above 9.4 GPa. Moreover, as shown in Supplementary Fig. 16, the states near the conduction band minimum (CBM) and the valence band maximum (VBM) are mainly contributed by S-3p, Cu1-3d, and Cu2-3d, manifesting that Cu1 framework and Cu2 soft lattices jointly dominate electron transport. The Cu2-3d has a decreasing contribution on VBM with increasing pressure, suggesting that Cu1 framework majorly serves as the conducting channel under high pressures.

3. Supplementary Figures and Tables

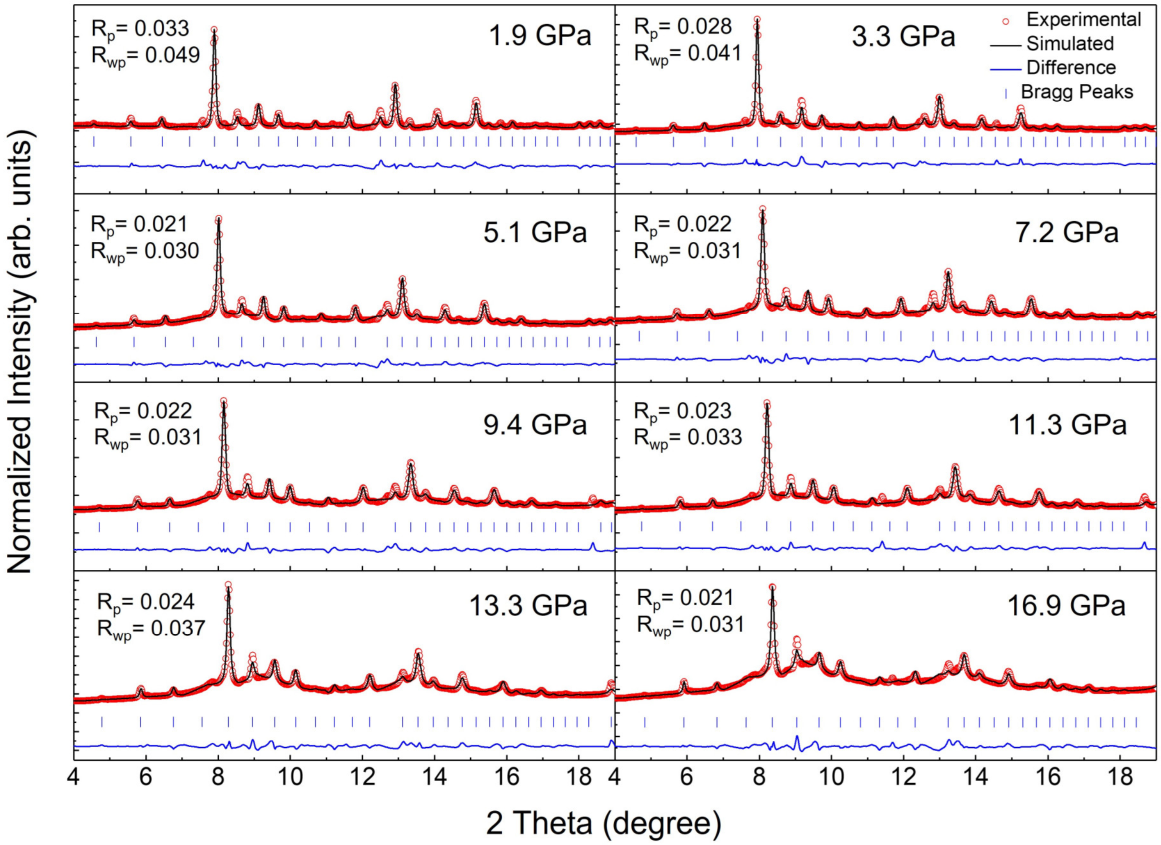

Supplementary Fig. 1. Rietveld refinement results of $Cu_{12}Sb_4S_{13}$ at selected pressures.

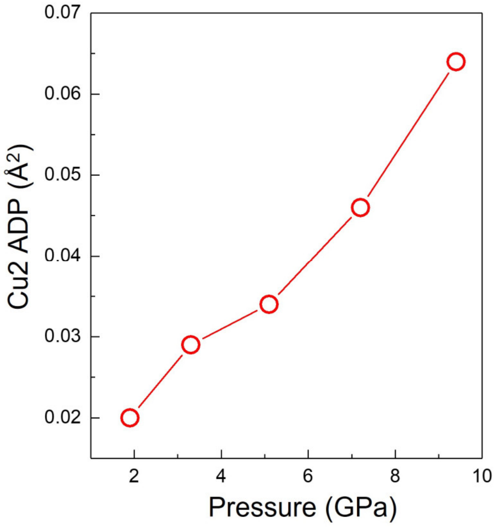

Supplementary Fig. 2. Cu2 atomic displacement parameters (ADP) of $Cu_{12}Sb_4S_{13}$ at different pressures. The ADP values are derived from the Rietveld refinement of synchrotron X-ray diffraction. The ADP values of Cu2 increase from 0.020 to 0.064 Å^2 during compression.

Supplementary Table 1. Crystal data of $Cu_{12}Sb_4S_{13}$ at selected pressures.

Pressure (GPa)	Space Group	*a* (Å)	*V* (Å^3)
0	*I-43m*	10.3678(7)	1114.45(2)
1.9	*I-43m*	10.2274(11)	1069.78(19)
3.3	*I-43m*	10.1614(10)	1049.19(17)
5.1	*I-43m*	10.0768(10)	1023.23(17)
7.2	*I-43m*	9.9782(11)	993.48(18)
9.4	*I-43m*	9.9024(12)	971.0(2)
11.3	*I-43m*	9.8412(14)	953.1(2)
13.3	*I-43m*	9.753(2)	927.6(4)
15.4	*I-43m*	9.6615(16)	901.9(3)
16.9	*I-43m*	9.612(2)	888.1(4)

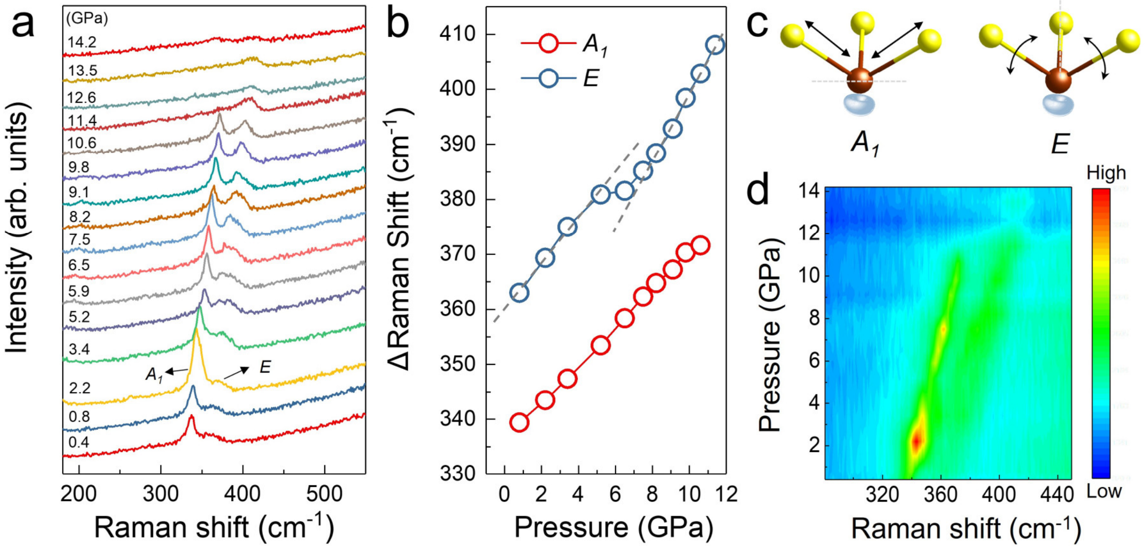

Supplementary Fig. 3. a Representative Raman spectra of $Cu_{12}Sb_4S_{13}$ at the selected pressures. **b** Experimental pressure dependence of the Raman-active modes of $Cu_{12}Sb_4S_{13}$. **c** The atomic displacement of representative Raman-active vibrational modes in $Cu_{12}Sb_4S_{13}$. **d** The corresponding 2D Raman intensity maps plot of pressure converted from **a**.

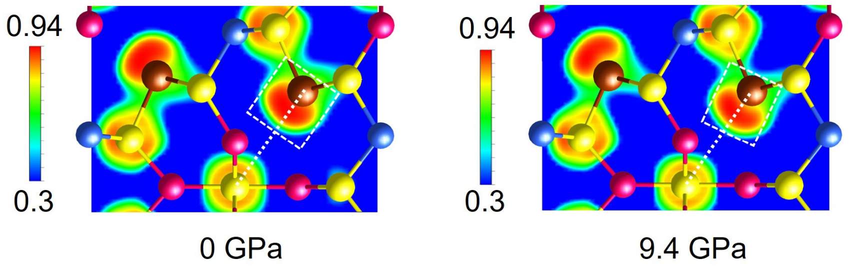

Supplementary Fig. 4. Valence electron density maps at ambient pressure and at 9.4 GPa. The ball-and-stick models with an isosurface value of ELF = 0.94 projects onto the (101) plane.

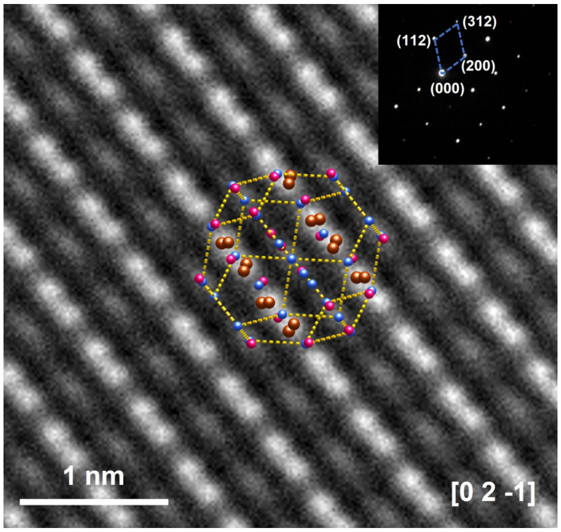

Supplementary Fig. 5. The HAADF images taken at ambient condition along the [0 2 -1] zone-axis. (Inset) Select area electron diffraction from this region.

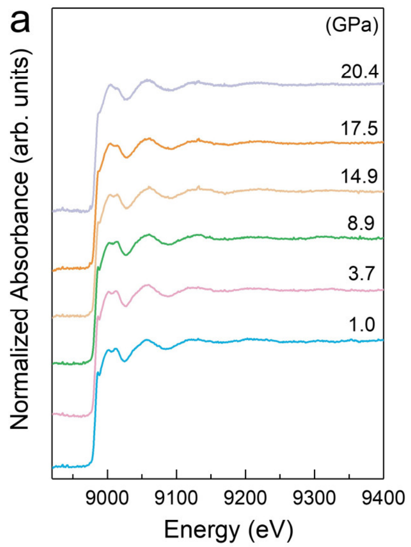

Supplementary Fig. 6. a The raw high-pressure XAS data of $Cu_{12}Sb_4S_{13}$ on the *K*-edge of Cu under pressure. **b** The *k*-weighted EXAFS spectra for $Cu_{12}Sb_4S_{13}$ as a function of pressure.

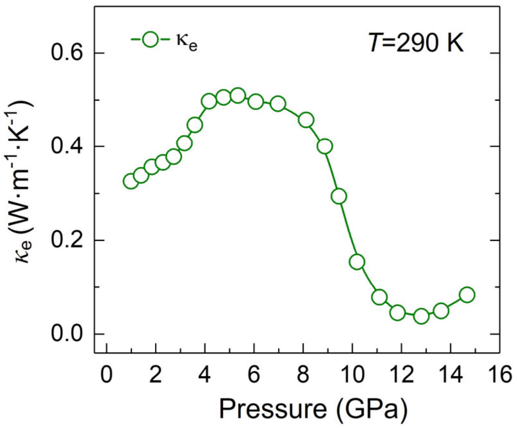

Supplementary Fig. 7. Pressure dependence of the electronic thermal conductivity (κ_e) at 290 K. The data were estimated from the pressure dependences of electrical resistivity (ρ).

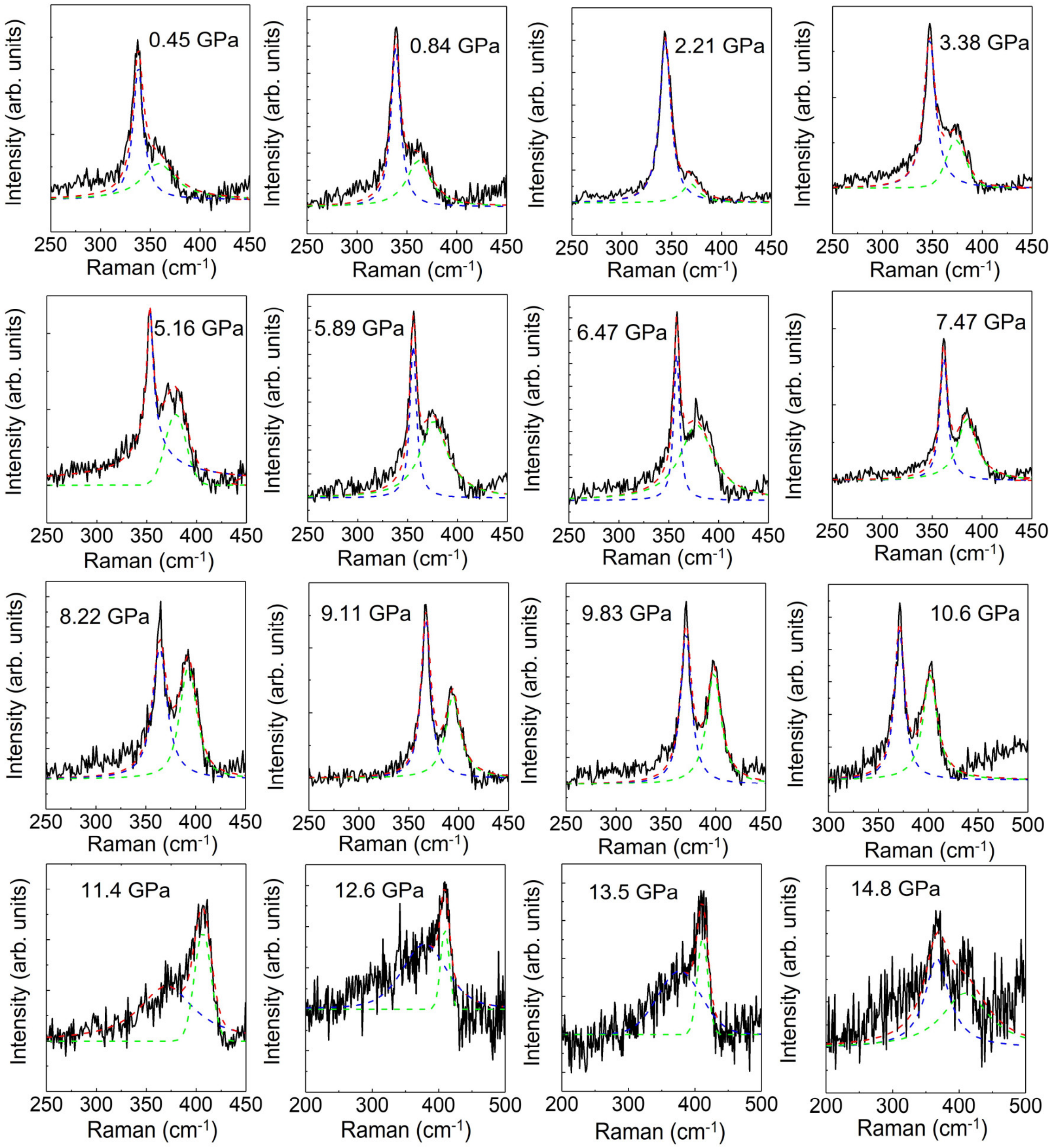

Supplementary Fig. 8. The Raman spectra at pressures up to 14.8 GPa with the fitting lines.

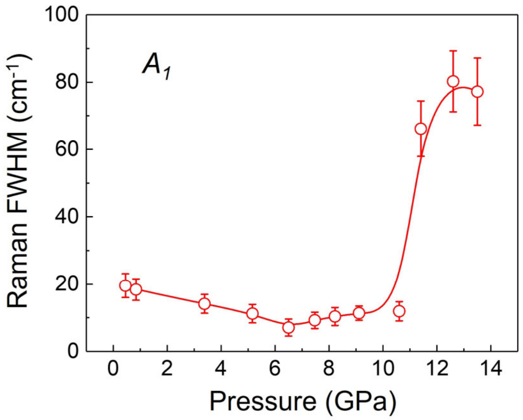

Supplementary Fig. 9. Pressure dependence of the FWHM of the Sb−S stretching optical mode A_1 in $Cu_{12}Sb_4S_{13}$.

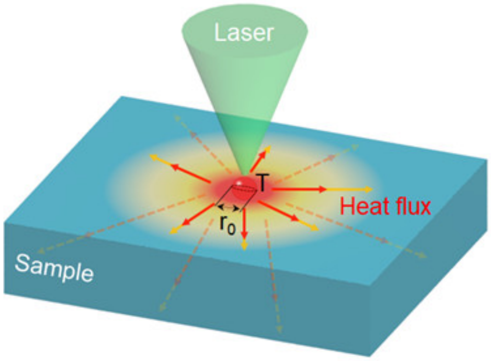

Supplementary Fig. 10. Schematic diagram for thermal conductivity measurement using Raman scattering method.

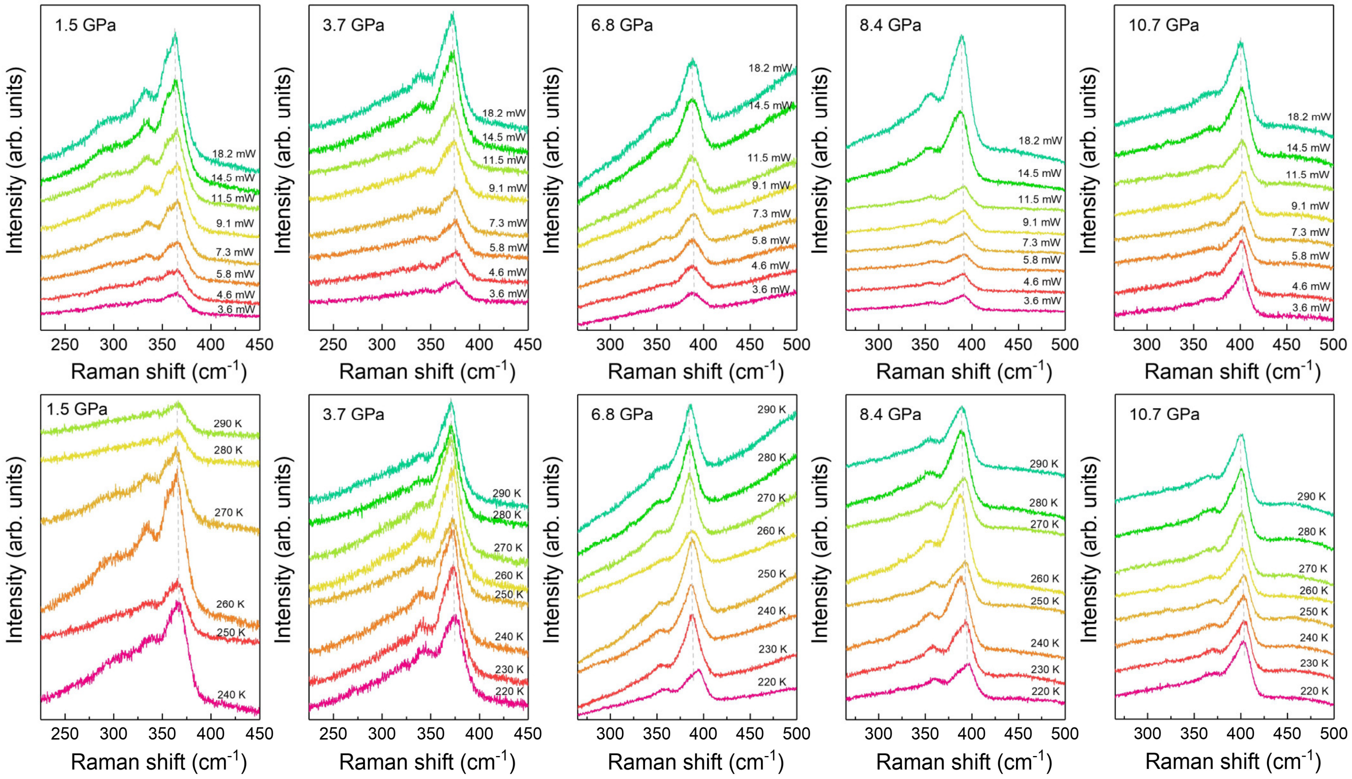

Supplementary Fig. 11. Raman spectra of the *E* mode at selected pressure measured as functions of temperature and laser power. The phonon frequencies were obtained by Lorentz fitting to the data.

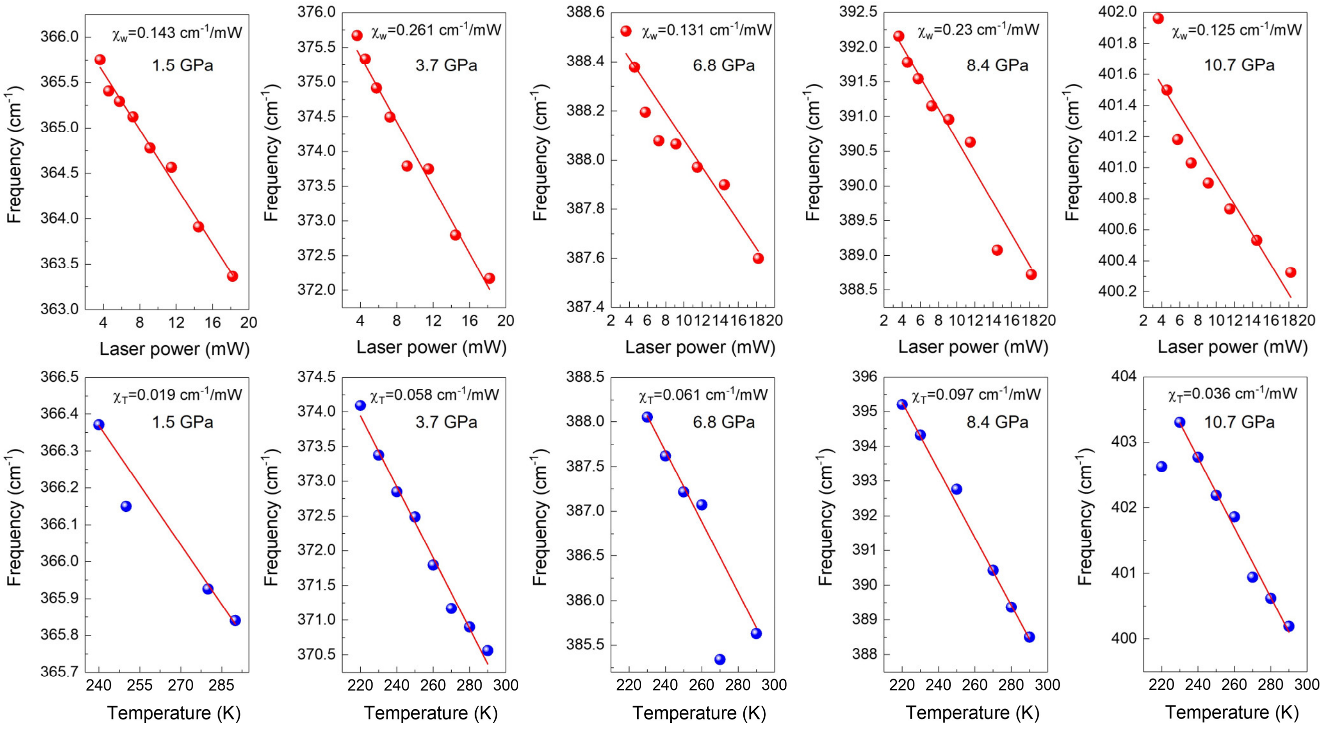

Supplementary Fig. 12. Frequencies of the *E* mode as functions of temperature and laser power at various pressures. χ_T and χ_W were obtained from the linear fitting to the data.

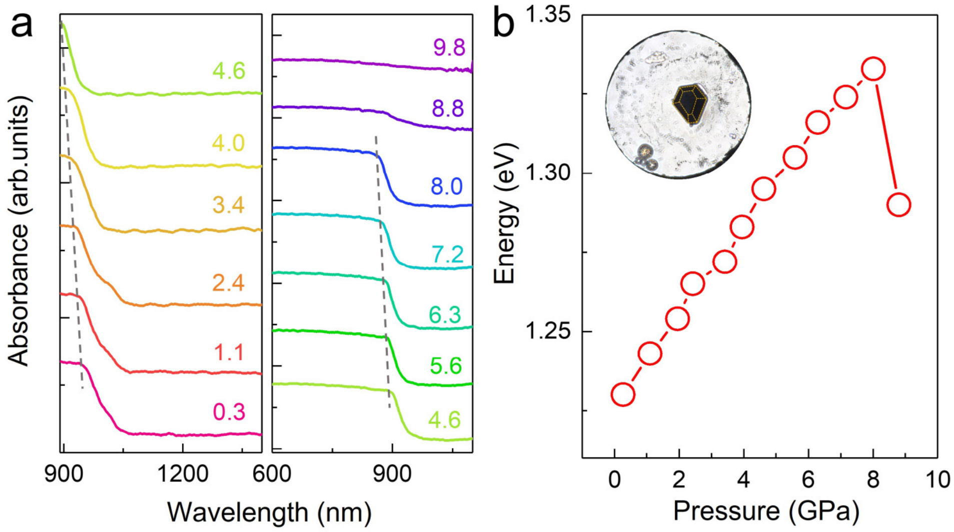

Supplementary Fig. 13. a Selected UV-Vis absorption spectra of single-crystal $Cu_{12}Sb_4S_{13}$ during compression. **b** Bandgap evolution of $Cu_{12}Sb_4S_{13}$ as a function of pressure.

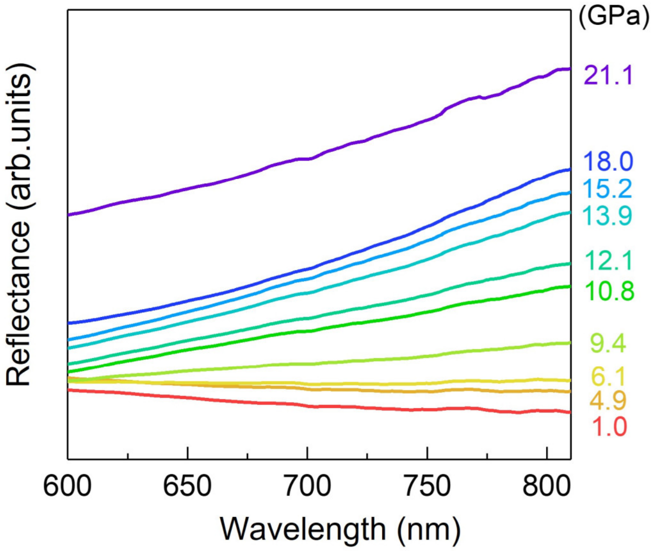

Supplementary Fig. 14. The optical reflectance of $Cu_{12}Sb_4S_{13}$ in near-infrared range at different pressures.

Supplementary Table 2. DFT relaxed crystal structures of $Cu_{12}Sb_4S_{13}$ at selected pressures.

Pressure (GPa)	*a* (Å)	*V* (Å^3)
0	10.38713	1120.694
1.9	10.22620	1069.406
3.3	10.16140	1049.205
5.1	10.07680	1023.217
7.2	9.97810	993.444
9.4	9.90270	971.093
11.3	9.84120	953.112
13.3	9.75300	927.715

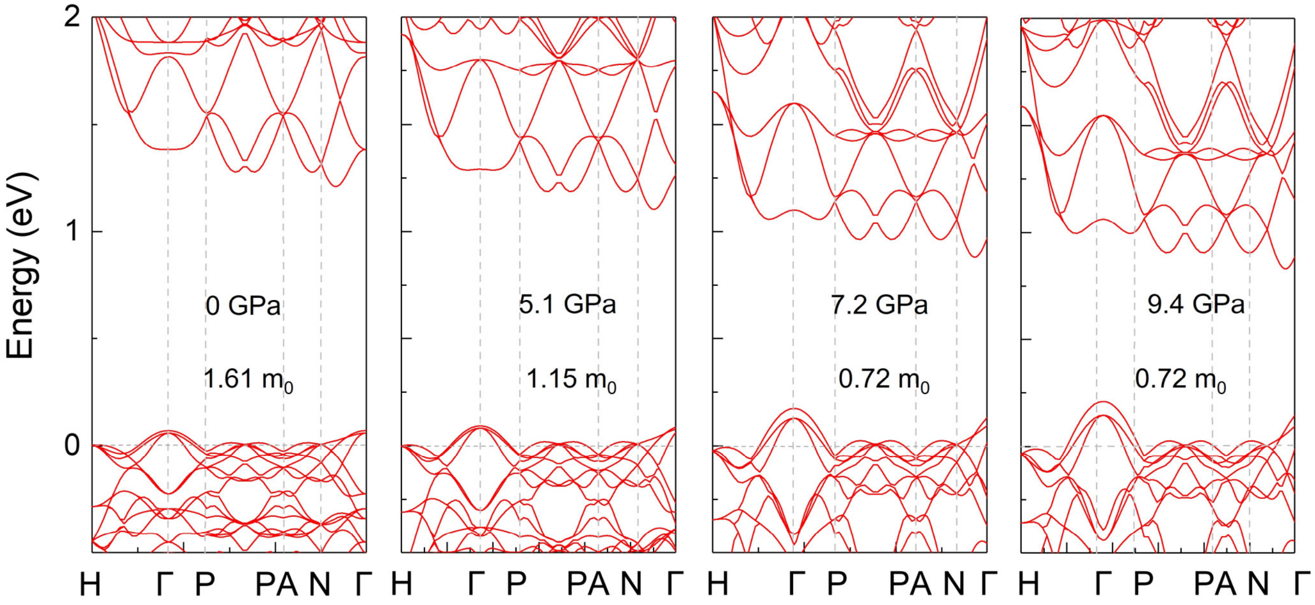

Supplementary Fig. 15. The calculated electronic structures at 0, 5.1, 7.2, and 9.4 GPa. Note that the fermi level lies inside the valence bands, indicating the p-type metalloid character of this compound. The effective masses of electron (m^*) derived from the electronic structures (Γ point) by the Supplementary Eq. (7). The m^* continually decrease upon compression.

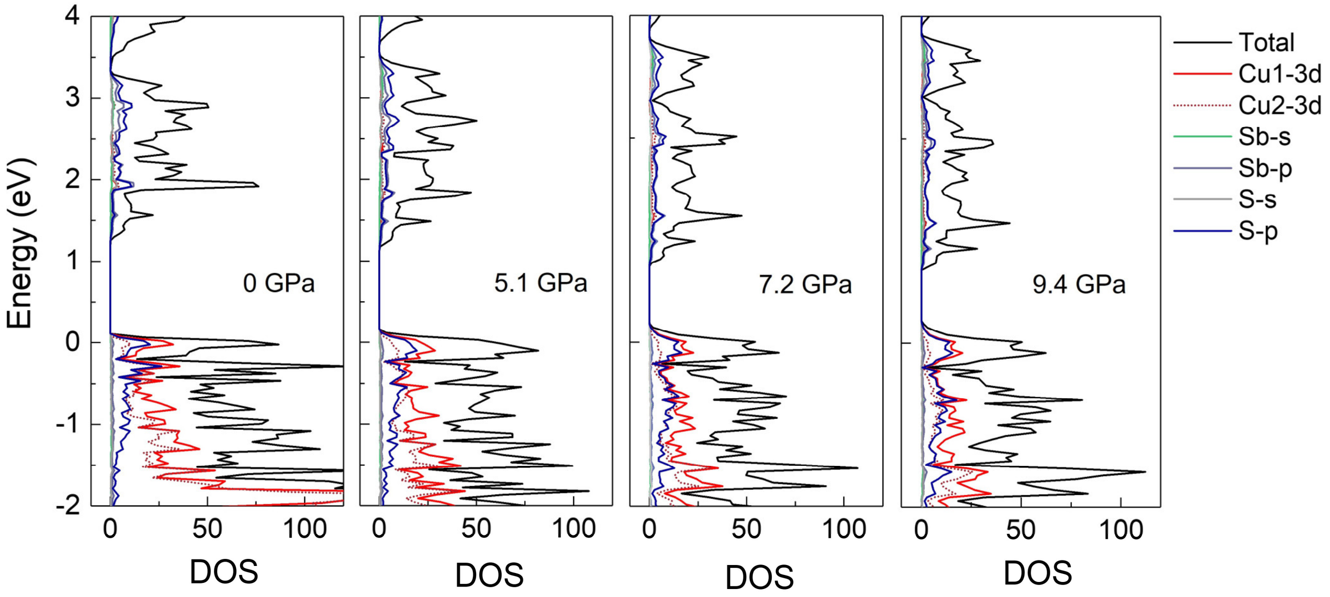

Supplementary Fig. 16. The calculated density of states of $Cu_{12}Sb_4S_{13}$ at 0, 5.1, 7.2, and 9.4 GPa.

Supplementary Table 3. Bader charges and DDEC bond orders of different atoms and atom pairs at different pressures. The bond orders of Cu1−S, Cu2−S, and Sb−S at ambient pressure are 0.63, 0.72, and 0.84, respectively, which indicate predominantly covalent bonding in the structure. The values of bond orders considerably increase under high pressures, suggesting the enhanced covalent bonding.

	Pressure (GPa)	Cu2	Cu1	Sb	average S
Bader charge	0	0.40	0.49	1.03	-0.75
	7.2	0.35	0.47	1.05	-0.72
	9.4	0.34	0.46	1.06	-0.70
	11.3	0.3	0.47	1.03	-0.71
	Pressure (GPa)	Cu2−Sb	Cu1−S	Cu2−S	Sb−S
Bond order	0	0.10	0.63	0.72	0.84
	7.2	0.20	0.70	0.78	0.86
	9.4	0.22	0.72	0.80	0.87
	11.3	0.25	0.75	0.84	0.83

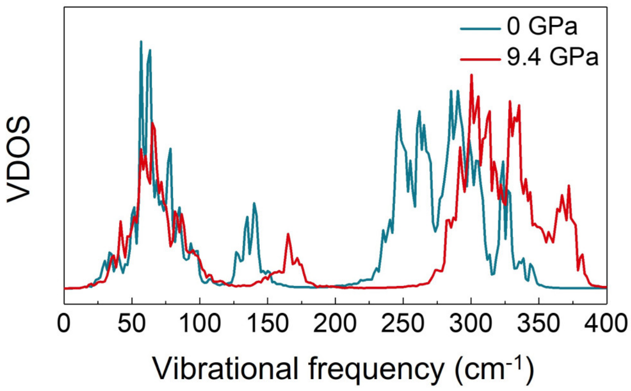

Supplementary Fig. 17. Vibrational density of states (VDOS) of $Cu_{12}Sb_4S_{13}$ tetrahedrites at 0 and 9.4 GPa.

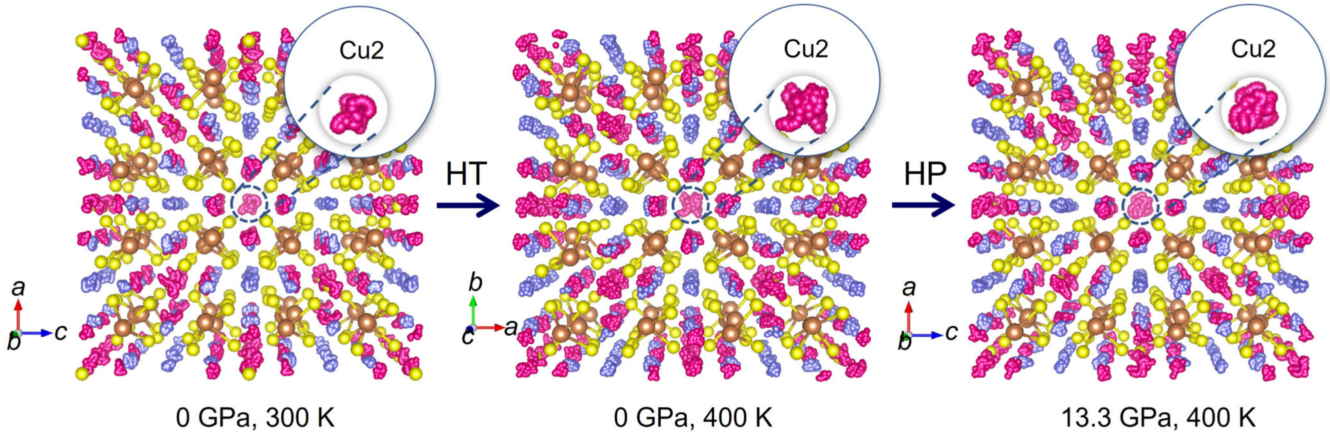

Supplementary Fig. 18. The trajectory of Cu atoms at selected pressures and temperatures.

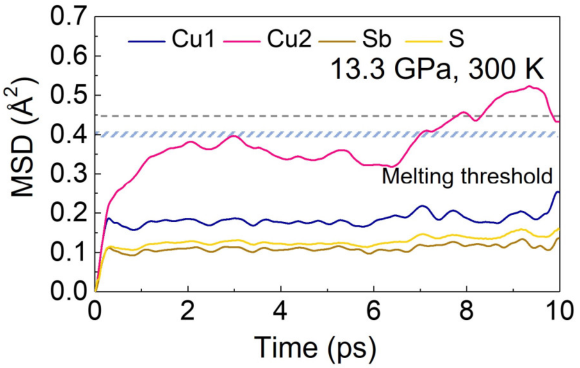

Supplementary Fig. 19. The time-dependent MSD under 13.3 GPa at 300 K. The dash and shade lines are average MSD and melting threshold MSD of Cu2 atoms, respectively.

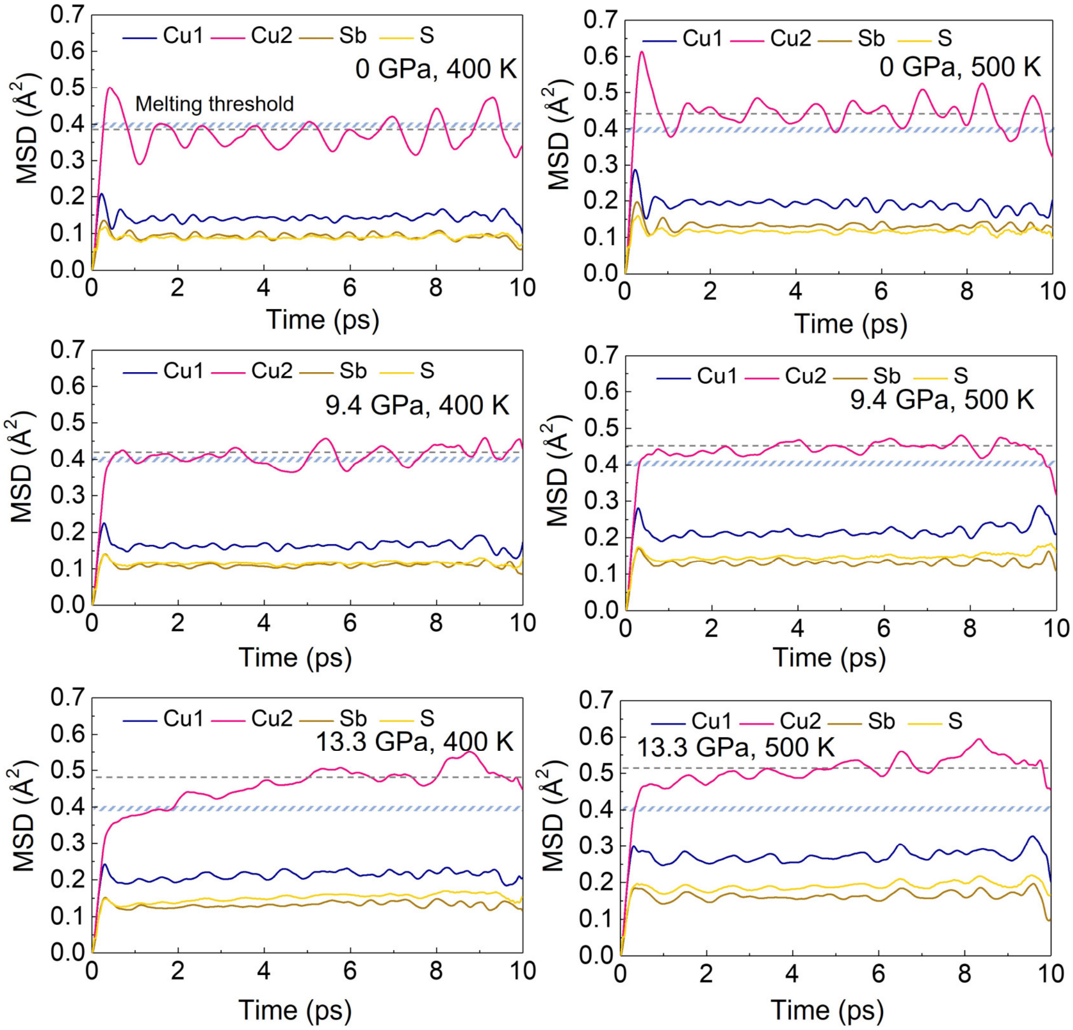

Supplementary Fig. 20. The time-dependent MSD at selected pressures at 400 K and 500 K. The dash and shade lines are average MSD and melting threshold MSD of Cu2 atoms, respectively.

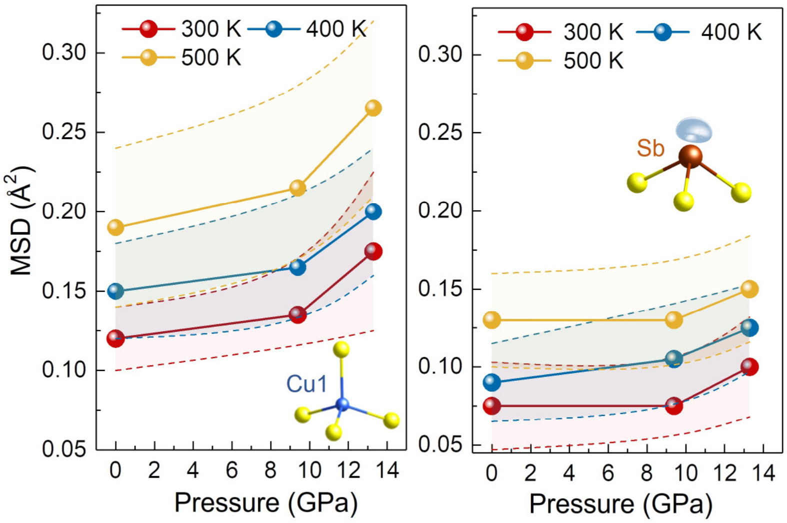

Supplementary Fig. 21. The MSD of Cu1 and Sb atoms in $Cu_{12}Sb_4S_{13}$ at different pressures and temperatures.

4. Supplementary References

1. Chen, X. et al. Structural transitions of 4: 1 methanol–ethanol mixture and silicone oil under high pressure. *Matter Radiat. at Extremes* **6**, 038402 (2021).

2. Prescher, C. & Prakapenka, V. B. DIOPTAS: a program for reduction of two-dimensional X-ray diffraction data and data exploration. *High Press Res* **35**, 223-230 (2015).

3. Rodríguez-Carvajal, J. FullProf. *CEA/Saclay*, (2001).

4. Wuensch, B. The crystal structure of tetrahedrite, $Cu_{12}Sb_4S_{13}$. *Z Kristallogr Cryst Mater* **119**, 437-453 (1964).

5. Baughman, R. H., Stafström, S., Cui, C. & Dantas, S. O. Materials with negative compressibilities in one or more dimensions. *Science* **279**, 1522-1524 (1998).

6. Sengers, J., Bolk, W. & Stigter, C. The thermal conductivity of neon between 25°C and 75°C at pressures up to 2600 atmospheres. *Physica* **30**, 1018-1026 (1964).

7. Cai, W. et al. Thermal transport in suspended and supported monolayer graphene grown by chemical vapor deposition. *Nano Lett.* **10**, 1645-1651 (2010).

8. Yan, R. et al. Thermal conductivity of monolayer molybdenum disulfide obtained from temperature-dependent Raman spectroscopy. *ACS Nano* **8**, 986-993 (2014).

9. Najmaei, S., Liu, Z., Ajayan, P. & Lou, J. Thermal effects on the characteristic Raman spectrum of molybdenum disulfide (MoS_2) of varying thicknesses. *Appl. Phys. Lett.* **100**, 013106 (2012).

10. Tan, G., Zhao, L. & Kanatzidis, M. G. Rationally designing high-performance bulk thermoelectric materials. *Chem. Rev.* **116**, 12123-12149 (2016).

11. Hofmeister, A. M. Pressure dependence of thermal transport properties. *Proc. Natl. Acad. Sci. U.S.A.* **104**, 9192-9197 (2007).

12. Chen, L. et al. Enhancement of thermoelectric performance across the topological phase transition in dense lead selenide. *Nat. Mater.* **18**, 1321-1326 (2019).

13. Kumar, D. P., Ren, M., Osipowicz, T., Mallik, R. C. & Malar, P. Tetrahedrite ($Cu_{12}Sb_4S_{13}$) thin films for photovoltaic and thermoelectric applications. *Sol Energy* **174**, 422-430 (2018).

14. Kresse, G. & Furthmüller, J. Efficient iterative schemes for ab initio total-energy calculations using a plane-wave basis set. *Phys. Rev. B* **54**, 11169 (1996).

15. Perdew, J. P., Burke, K. & Ernzerhof, M. Generalized gradient approximation made simple. *Phys. Rev. Lett.* **77**, 3865 (1996).

16. Kresse, G. & Hafner, J. Ab initio molecular-dynamics simulation of the liquid-metal–amorphous-semiconductor transition in germanium. *Phys. Rev. B* **49**, 14251 (1994).

17. Nosé, S. A unified formulation of the constant temperature molecular dynamics methods. *J. Chem. Phys.* **81**, 511-519 (1984).

18. Peccerillo, E. & Durose, K. Copper–antimony and copper–bismuth chalcogenides—Research opportunities and review for solar photovoltaics. *MRS. Energy. Sustain.* **5**, 13 (2018).

19. Bu, K. et al. Pressure-Regulated Dynamic Stereochemical Role of Lone-Pair Electrons in Layered Bi_2O_2S. *J. Phys. Chem. Lett.* **11**, 9702-9707 (2020).
